# Continued stabilization of trabecular metal tibial monoblock total knee arthroplasty components at 5 years—measured with radiostereometric analysis

**DOI:** 10.3109/17453674.2011.645196

**Published:** 2012-02-08

**Authors:** David A J Wilson, Glen Richardson, Allan W Hennigar, Michael J Dunbar

**Affiliations:** ^1^School of Biomedical Engineering; ^2^Department of Surgery, Dalhousie University, Halifax, Canada

## Abstract

**Background and purpose:**

The trabecular metal tibial monoblock component (TM) is a relatively new option available for total knee arthroplasty. We have previously reported a large degree of early migration of the trabecular metal component in a subset of patients. These implants all appeared to stabilize at 2 years. We now present 5-year RSA results of the TM and compare them with those of the NexGen Option Stemmed cemented tibial component (Zimmer, Warsaw IN).

**Patients and methods:**

70 patients with osteoarthritis were randomized to receive either the TM implant or the cemented component. RSA examination was done postoperatively and at 6 months, 1 year, 2 years, and 5 years. RSA outcomes were translations, rotations, and maximum total point motion (MTPM) of the components. MTPM values were used to classify implants as “at risk” or “stable”.

**Results:**

At the 5-year follow-up, 45 patients were available for analysis. There were 27 in the TM group and 18 in the cemented group. MTPM values were similar in the 2 groups (p = 0.9). The TM components had significantly greater subsidence than the cemented components (p = 0.001). The proportion of “at risk” components at 5 years was 2 of 18 in the cemented group and 0 of 27 in the TM group (p = 0.2).

**Interpretation:**

In the previous 2-year report, we expressed our uncertainty concerning the long-term stability of the TM implant due to the high initial migration seen in some cases. Here, we report stability of this implant up to 5 years in all cases. The implant appears to achieve solid fixation despite high levels of migration initially.

Swedish Knee Arthoplasty Register data suggest that uncemented tibial components have worse survivorship than cemented components (Knutson and Robertsson 2010). However, more recent data from Australia have suggested that modern uncemented designs have a similar cumulative revision rate to that of cemented components (Graves et al. 2004). With recent improvements in biomaterials, the potential for improved longevity using uncemented implants is once again being explored. Trabecular metal (Zimmer, Warsaw, IN) is a new material available for use in uncemented total knee arthroplasty. It is a porous biomaterial with morphology and mechanical properties resembling those of trabecular bone (Bobyn et al. 1999a,b, Zardiackas et al. 2001, Levine et al. 2006, Balla et al. 2010). In a previous paper, we presented the 2-year implant migration results of the trabecular metal tibial monoblock component using radiostereometric analysis (RSA) (Dunbar et al. 2009). The results of that study showed high initial migration in 9 out of 28 of the tibial components, with apparent stabilization occurring after 1 year in all cases. These results reflected the results of a similar RSA study on the same implant, performed at another center (Henricson et al. 2008). In addition, in the 2-year report we also documented apparent deformation of the tibial base plate, which occurred in 5 of the cases of high migration (Dunbar et al. 2009). The implications of the high degree of migration and plate deformation for long-term survival are not known. In a subset of the patients enrolled in the study, we also performed RSA for measurement of inducible displacement between 2 and 4 years. The results of this study showed that the trabecular metal component had the lowest inducible displacement ever reported in the literature, indicating excellent stability (Wilson et al. 2010). The results of these 3 studies supported the hypothesis that these components were achieving adequate bone in-growth for long-term survival (Henricson et al. 2008, Dunbar et al. 2009, Wilson et al. 2010). However, longer follow-up is necessary to determine whether the early stability of these implants is durable. In this paper, we present the 5-year longitudinal RSA results from the original cohort of patients randomized to receive either the Nexgen LPS monoblock (trabecular metal) tibial component (Zimmer) or the cemented NexGen Option Stemmed tibial component (Zimmer) (Dunbar et al. 2009).

## Patients and methods

70 subjects with severe osteoarthritis were randomized to receive either the Nexgen LPS monoblock (trabecular metal) tibial component (Zimmer, Warsaw, IN) or the cemented NexGen Option Stemmed tibial component (also Zimmer). Surgery was performed by 4 experienced consultant knee surgeons using a standardized protocol: posterior cruciate ligament resection, patellar resurfacing with a cemented inlay component, cementing of the femoral component, and RSA marker placement of 0.8mm beads. 4–6 tantalum markers were placed around the periphery of the polyethylene component; a median of 10 tantalum markers were inserted into the proximal tibia. The postoperative protocol was standardized with the use of continuous passive motion as tolerated, and patients were allowed full weight bearing immediately. No drains were used.

At the 5-year postoperative time point, patients were contacted and asked if they would consent to continued participation in the study. Written informed consent was obtained from patients who had verbally agreed to participate, when they arrived for their 5-year follow-up RSA examination. This was in accordance with requirements of our Institutional Ethics Review Board.

The knee was placed above a uniplanar calibration box (Medis Specials, Leiden, the Netherlands) and simultaneous digital stereo radiographs were taken with the X-ray tubes oriented obliquely. Western Ontario and McMaster University Osteoarthritis Index (WOMAC) scores were obtained at 5 years. Full details of the original study design can be found in the previously published article (Dunbar et al. 2009). The original 2-year study was registered at ClinicalTrials.gov (NCT00405379).

### Radiostereometric analysis

RSA data analysis was performed using commercial software (MB-RSA; MEDIS, Leiden, the Netherlands). All translations and rotations were calculated to comply with the standards presented by Valstar et al. (2005). Rigid-body translations and rotations of the prosthesis were calculated about a coordinate system centered at the volumetric center of the implant, with axes aligned with the anatomical directions. The maximum total point motion of the tibial component for each case was calculated using fictive markers to standardize the calculations in cases where the prosthesis bead placement was not uniform for all subjects (Nilsson and Karrholm 1993, Nilsson et al. 1999, Valstar et al. 2005). The limit of rigid body fitting was a maximum of 0.2 mm for the tibial segment and 0.2 mm for the prosthesis segment. The condition number did not exceed 40 at any follow-up, indicating adequate distribution of beads in the rigid body (Valstar et al. 2005). The accuracy of the RSA system was assessed with a standard phantom study protocol. Accuracy was represented as half of the average width of the 95% prediction interval in a regression analysis of true and measured translations of a phantom. Precision was evaluated with double examination analysis, and represented as the 95% confidence interval of the measurements from 11 double clinical examinations performed at the postoperative follow-up. The marker configuration model-based RSA technique (Kaptein et al. 2005) was used to solve the occluded marker problem in cases where less than 3 beads were matched in an examination at follow-up. Marker configuration models were also used to test for bead loosening and possible deformation of the trabecular metal base plate.

At the 2-year follow-up, implants were classified as being “stable” (< 0.2 mm MTPM between 1 and 2 years' follow-up) or as being “at risk” of early aseptic loosening (> 0.2 mm MTPM between 1 and 2 years' follow-up) (Ryd et al. 1995). Implants categorized as “at risk” at 2 years were re-evaluated, and if found to have less then 0.3 mm of motion between 2 and 5 years, they were considered to have stabilized. Implants considered to be “at risk” at 2 years with greater than 0.3 mm of motion between 2 and 5 years were still considered to be “at risk”. Implants classified as being “stable” at 2 years but which showed more than 0.3 mm of motion between 2 and 5 years were classified as being “at risk”. 0.3 mm was chosen as the threshold for migration over 3 years by extrapolating the 0.2 mm between 2-year follow-up periods, originally described as the modified continuous migration (MCM) criteria by Ryd et al. (1995).

### Statistics

Analysis of variance was used to test for differences in age, body mass index (BMI), and subjective measures between implant groups. Maximum total point motion measurements were not normally distributed; therefore, the Kruskal-Wallis test was used to investigate differences in maximum total point motion between implant groups at 5 years. Analysis of variance was used to test for differences in translations and rotations between groups at 5 years. Fisher's exact test was used to investigate differences in proportions of implants found to be “at risk” between groups, and Wilson's procedure was used to calculate the 95% confidence intervals of this proportion. There were 8 primary outcome metrics (maximum total point motion, 3 translations, 3 rotations, and proportion of implants at risk). A Bonferroni correction for multiple comparisons was used, giving a significance level of p < 0.006.

## Results

### Standard outcomes and follow-up

All standard outcome measures for the 45 patients in this study are given in Table 1. Originally, 37 patients were randomized to receive the trabecular metal component and 33 received the cemented component. Age and BMI were similar in the 2 groups at the time of admission. There were no differences in subjective measures (WOMAC) between implant groups at any follow-up point.

**Table 1.** Subject demographics and WOMAC scores. Only matched WOMAC data from earlier follow-ups are included. Values are mean (SD)

	Trabecular metal	Cemented	p-value
	(n = 27)	(n = 18)	(ANOVA)
Sex, M/F	10/17	8/10	
Age	60 (8)	61 (9)	0.7
Body mass index	32 (5)	34 (5)	0.2
WOMAC preoperatively	51 (20)	44 (18)	0.2
WOMAC at 6 months	22 (23)	18 (20)	0.5
WOMAC at 12 months	15 (16)	14 (22)	0.9
WOMAC at 24 months	13 (16)	15 (14)	0.6
WOMAC at 60 months	18 (18)	19 (18)	0.8

**Figure 1.**
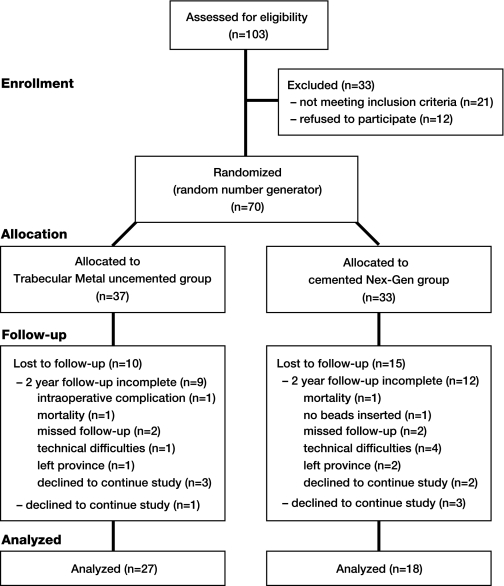
Consolidated Standards of Reporting Trials (CONSORT) flow diagram.

For the 5-year follow-up, 45 patients could be reached and were willing to participate. There were 27/28 of the trabecular metal subjects and 18/21 cemented subjects with 2-year data recruited. The reasons for loss to follow-up for the other 25 patients are documented in Figure 1 and it happened mainly in the first two years of follow-up. Of the patients with a high degree of prosthesis migration in the original study, all 9 were recruited to the 5-year follow-up. All but 3 of the patients originally recruited to the study are still being followed by the operative surgeon, and there have been no revisions to date. The three patients who were exceptions had left the province.

### RSA results

The accuracy of the RSA system was 0.025 mm, 0.025 mm, 0.063 mm, and 0.026 mm for the x-, y-, and z- directions and maximum total point motion, respectively. The precision of the RSA system was 0.07 mm, 0.07 mm, and 0.11 mm for x-, y-, and z- translations, 0.16°, 0.15°, and 0.12° for x-, y-, and z- rotations, and 0.10 mm for maximum total point motion.

At the 2-year follow-up, there were no subjects “at risk” in the trabecular metal group (0/28). At the 5-year follow-up, none of the trabecular metal components were found to be “at risk”. At the 2-year follow-up, there were 4 subjects “at risk” in the cemented group (4/21). At the 5-year follow-up, 1 of the cemented components “at risk” was lost to follow-up, 2 stabilized (0.09 mm and 0.07 mm of movement between 2 and 5 years) and 1 continued to migrate (0.35 mm of movement between 2 and 5 years). 1 of the cemented implants found to be stable at 2 years migrated an additional 0.42 mm and was classified as being “at risk”. However, retrospectively, this implant had somewhat anomalous 2-year data (0.64 mm, 0.45 mm, and 0.87 mm at 1, 2, and 5 years), where the difference between 1 year and 5 years would make the implant appear stable (0.23 mm in 4 years) but the difference between 2 years and 5 years would make the implant appear to be continuously migrating (0.42 mm in 3 years). The condition number and mean errors for this subject were within normal limits. All other implants that were found to be stable at 2 years migrated less than 0.3 mm between the 2- and 5-year follow-up, and were still classified as stable. The proportion of “at risk” components at 5 years was 2 of 18 (0.11, 95% CI: 0.03–0.33) in the cemented group and was 0 of 27 (95% CI: 0.0–0.13) in the trabecular metal group (p = 0.2).

**Figure 2.**
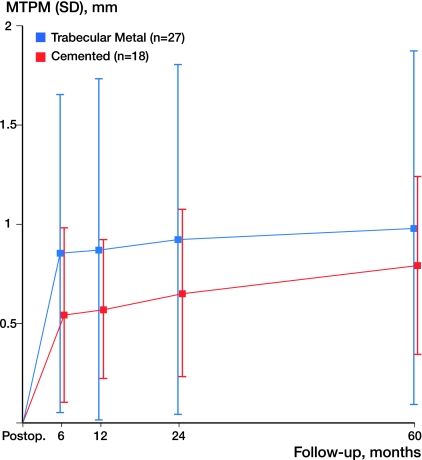
Maximum total point motion (MTPM) (mean and standard deviation) according to the duration of follow-up in the trabecular metal and cemented groups. Lines joining data points do not indicate continuous measurement but are included to show the pattern of migration.

**Figure 3.**
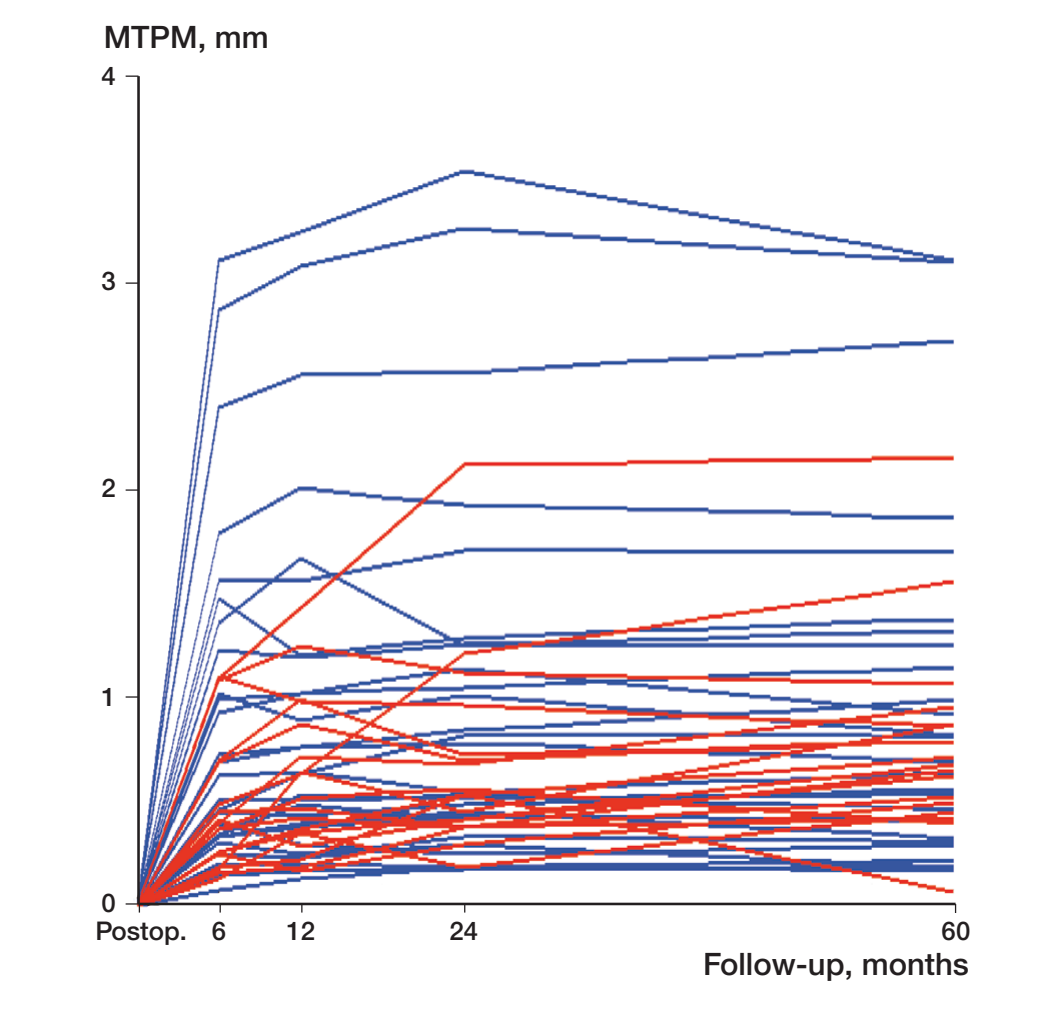
Individual maximum total point motion (MTPM) according to the duration of follow-up in the trabecular metal group (blue) and the cemented group (red). Lines joining data points do not indicate continuous measurement but are included to show the pattern of migration.

At the 5-year follow-up, there was no difference in MTPM between the cemented group and the trabecular metal group (p = 0.9) (Table 2 and Figure 2). MTPM values for individual subjects are shown in Figure 3. Compared to the cemented components, the trabecular metal tibial components had more subsidence (p = 0.001) (Table 2).

**Table 2.** Summary of radiostereometric analysis (RSA) data by group and follow-up

	6-month follow-up		12-month follow-up		24-month follow-up		60-month follow-up		p-value**^a^**
	TM	Cemented	TM	Cemented	TM	Cemented	TM	Cemented	
	(n=30)	(n=24)	(n=29)	(n=23)	(n=28)	(n=21)	(n=27)	(n=18)	
Mean maximum total point motion (range)	0.85 (0.07–3.11)	0.54 (0.12–1.99)	0.87 (0.12–3.25)	0.57 (0.16–1.87)	0.92 (0.06–3.40)	0.65 (0.18–2.12)	0.98 (0.16–3.11)	0.79 (0.06–2.16)	0.9
Lateral/medial translation (SD)	–0.02 (0.13)	0.01 (0.14)	–0.02(0.13)	–0.00(0.14)	–0.01 (0.14)	0.02 (0.16)	–0.02 (0.16)	0.01 (0.26)	0.7
Superior/inferior translation (SD)	–0.34 (0.31)	0.00 (0.08)	–0.34 (0.34)	–0.01 (0.11)	–0.35 (0.35)	–0.03 (0.10)	–0.38 (0.37)	–0.04 (0.11)	0.001
Anterior/posterior translation (SD)	0.02 (0.12)	0.02 (0.29)	0.03 (0.13)	0.06 (0.18)	0.02 (0.12)	0.09 (0.18)	0.01 (0.12)	0.08 (0.29)	0.3
Anterior/posterior tilt (SD)	–0.33 (0.73)	0.02 (0.43)	–0.40 (0.80)	–0.02 (0.30)	–0.39 (0.80)	–0.05 (0.40)	–0.45 (0.90)	–0.11 (0.26)	0.1
Internal/external rotation (SD)	0.19 (0.38)	–0.15 (0.47)	0.15 (0.39)	–0.14 (0.44)	0.19 (0.45)	–0.08 (0.60)	0.17 (0.45)	0.04 (0.82)	0.5
Varus/valgus tilt (SD)	–0.05(0.72)	0.07 (0.32)	–0.11 (0.73)	0.06 (0.32)	–0.10 (0.75)	0.06 (0.33)	–0.12 (0.73)	–0.02 (0.20)	0.6

**^a^**5-year results only

At the 2-year follow-up, 9 subjects in the trabecular metal group had shown very high migration in the first 6 months postoperatively (> 1.0 mm). All of these implants migrated less than 0.2 mm between 1 and 2 years and were not considered to be “at risk” At 5 years, all of these implants continued to show stability with an average change in MTPM of 0.10 mm over the 3 years between follow-ups.

Using the marker configuration models, independent bead motion was detected in 5 of the high-migration cases, between the postoperative and 6-month follow-up examinations. The error of fit of the model marker was reduced dramatically (0.103 mm to 0.0466 mm on average) when the bead in question was removed from the analysis. The position of the bead with respect to that of the bead in the model was inferior, indicating that the motion was not due to loose beads, which would have traveled radially. The magnitude of independent bead motion was from 0.3 mm to 0.5 mm in all 5 cases. At the 5-year follow-up, there was no change in the position of the independently moving bead—indicating that the deformation of the base plate was permanent.

## Discussion

In the original 2-year report on these patients, we expressed our uncertainty concerning the long-term stability of the trabecular metal tibial implant due to the high initial migration seen in some cases. Subsequent to this, we have found stability of this implant up to 5 years and migration below the level of detection of our system in all cases. This implant appears to achieve solid fixation despite high initial levels of migration.

The one cemented implant that was originally classified as being stable at 2 years but which appeared to be continuously migrating between 2 and 5 years was an unusual finding. We believe that due to random error, the MTPM of this subject was underestimated at 2 years, leading to a spurious finding of continuous migration between 2 and 5 years. However, this patient will continue to be monitored closely in the clinic.

The mechanism that results in high early migration and subsequent stabilization is unknown. Previous work (Bellemans 1999) suggested that uncemented components can migrate extensively early after implantation and still form a robust interface with bone. However, migrations of such high magnitude (> 2.5 mm) are more typically associated with continuous migration (Grewal et al. 1992). In our work, all of the initially high migration stabilized by 1 year, suggesting that long-term stability can be achieved even in cases of quite extensive migration. Furthermore, our previous inducible displacement study on this patient group included subjects with a high degree of early migration, and all showed a low degree of inducible displacement (Wilson et al. 2010). Low inducible displacement suggests that bone ingrowth has occurred, and is inconsistent with fixation characterized by fibrous encapsulation. One possible explanation for this novel behavior may be related to the mechanical properties of the trabecular metal material. Due to its low elastic modulus, the trabecular metal monoblock component may deform, allowing areas of the implant that were well fixed initially to remain stable and achieve ingrowth of bone instead of lifting off as the contralateral side sinks into the bone. In support of this, we did measure permanent deformation of the trabecular metal tibial base plate in some of the high-migration cases (Dunbar et al. 2009).

The most important limitation of this study was the small sample size. With only 37 patients randomized to the trabecular metal arm of the study, and of these only 27 being willing to participate in the 5-year follow-up, our ability to draw firm conclusions about the risk of revision in patients with this implant is somewhat limited. Another limitation is the modular nature of the cemented components. As the polyethylene was the part of the implant marked with RSA beads, any motion of the polyethylene component with respect to the tibial base plate would be detected as migration of the implant. It is possible that this contributed to the migration detected in some of the cemented cases. However, the monoblock construction of the trabecular metal implant makes motion between the polyethylene and base plate unlikely and less of a concern in that group.
